# Embodying Emotional Disorders: New Hypotheses about Possible Emotional Consequences of Motor Disorders in Parkinson's Disease and Tourette's Syndrome

**DOI:** 10.5402/2011/306918

**Published:** 2011-07-09

**Authors:** Martial Mermillod, Nicolas Vermeulen, Sylvie Droit-Volet, Isabelle Jalenques, Franck Durif, Paula Niedenthal

**Affiliations:** ^1^Laboratoire de Psychologie Sociale et Cognitive (LAPSCO) and CNRS, Université Blaise Pascal, Clermont Université, BP 10448, 63000 Clermont-Ferrand, France, UMR, and CNRS 6024, 34 avenue Carnot, 63037 Clermont-Ferrand Cedex, France; ^2^Institut Universitaire de France, France; ^3^Research Unit for Emotion, Cognition, and Health, Psychology Department, Université Catholique de Louvain (UCL), 10 Place du Cardinal Mercier, B-1348 Louvain-la-Neuve, Belgium; ^4^Fund for Scientific Research (FRS-FNRS), Belgium; ^5^Service de Psychiatrie A (EA 3845), CHRU Clermont-Ferrand Gabriel-Montpied, 58 Boulevard Montalembert, 63003 Clermont Ferrand, France; ^6^Service de Neurologie A (EA 3845), CHRU Clermont-Ferrand Gabriel-Montpied, 58 Boulevard Montalembert, 63003 Clermont Ferrand, France

## Abstract

Parkinson's disease (PD) and Tourette's syndrome (TS) lead to important motor disorders among patients such as possible facial amimia in PD and tics in Tourette's syndrome. Under the grounded cognition framework that shows the importance of motor embodiment in emotional feeling (Niedenthal, 2007), both types of pathology with motor symptoms should be sufficient to induce potential impairments for these patients when recognizing emotional facial expressions (EFE). In this opinion paper, we describe a theoretical framework that assumes potential emotional disorders in Parkinson's disease and Tourette's syndrome based on motor disorders characterizing these two pathologies. We also review different methodological barriers in previous experimental designs that could enable the identification of emotional facial expressions despite emotional disorders in PD and TS.

## 1. Grounded Cognition Theory

**Figure 1:**
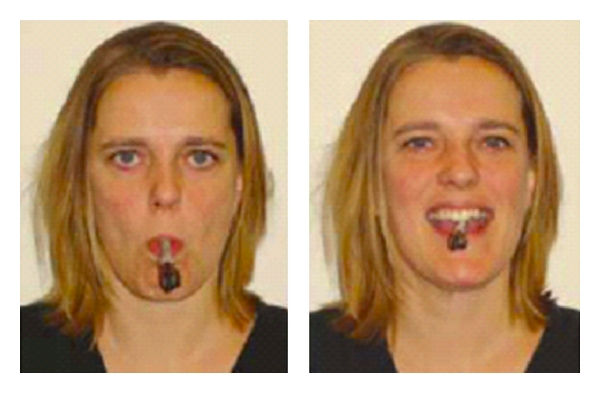
Experimental procedure used by Strack et al. [5].

A prominent view of cognitive and emotional processes assumes that conceptual knowledge emerges from bottom-up perceptual processes to conceptual and a modal symbolic systems [1]. An alternative view has been proposed under the theoretical framework of theories of grounded cognition [2–4] suggesting that associative, limbic, and sensorimotor functional components are automatically reactivated for access to specific conceptual knowledge. This theoretical view assumes that disturbing motor processing can induce recognition impairment at the perceptual level. For instance, Strack et al. [5] have shown that participants holding a pen between their lips to inhibit smiling, or holding the pen between their teeth to facilitate smiling (Figure 1), produced significant emotional modulation with respect to the funniness of cartoons (depending on the group of inhibited facial muscles). In what follows, we argue that consistent with the grounded cognition theory, motor disorders characterizing different psychopathologies (Parkinson's disease and Tourette's syndrome) could be sufficient to induce disturbances in emotion processing.

Different neural structures have been identified as playing an important role in grounded cognition. For example, these type of links between motor systems and action understanding had previously been developed in the literature on mirror neurons, first shown in monkeys [6, 7] and more recently among humans where premotor and parietal areas were identified as important neural structures for mirror neuron system [6–8]. Cortical mirror neurons were interpreted as automatic neural processes involved in basic and implicit comprehension of action by other individuals [6, 7]. In a similar line of research, Wicker et al. [9] have shown that mirror neurons at the level of the insula may support both the feeling and the perception of disgust. This finding suggests that mirror neuron areas dedicated to the functional integration of not only motor but also emotional processes could be widely spread across different high-level cortical areas.

Among these high-level cortical structures, somatosensory cortices seem to be a good candidate for the *feeling* of emotions. For example, the right hemisphere cortex seems to be particularly involved in the processing of recognition of emotional expressions [10], and lesion studies specifically implicate somatosensory cortex. In an attempt to shed further light on the function of these anatomical structures, Adolphs et al. [11] carried out a quantitative study involving a detailed analysis of 108 subjects with focal brain lesions. Results show a major implication of right somatosensory cortices, including the right anterior supramarginal gyrus, the lower sector of S-I and S-II, the insula, and the left frontal operculum for recognizing the six basic emotional facial expressions. To a lesser extent, and consistent with previous studies [10], lesions of the right visual-related cortices also induced a deficit in recognizing specific facial expressions and especially fearful expressions. Moreover, Adolphs et al. [10] found complementary results showing that lesions that include the right inferior parietal cortex induced impairments in the recognition of negative emotions (especially fear and sadness). Consequently, they proposed that right somatosensory cortex generate a representation of the feeling state, which is sometimes used for recognition [12]. It is important to note that the structures involved in *recognizing* basic emotional facial expressions seem slightly different from those involved in the *conceptual knowledge* of the emotions showing the involvement of right somatosensory-related cortices (including S-I and S-II), the insula, and supramarginal gyrus. Access to conceptual knowledge was investigated by the authors [11] by means of a task requiring the sorting of EFE photographs into piles according to the similarity of the emotion displayed. It can be argued that such a task does not necessarily require conceptual knowledge; rather, the assessment of perceptual similarity could be sufficient to perform the given task. These differences between the goals and functions of somatosensory cortices versus parietal or temporal cortical areas illustrate the difference between the feeling of and the perceptual recognition of a specific emotional expression. Further studies in psychology and cognitive neuroscience have to carefully differentiate between these psychological tasks. We will further discuss these differences in the methodological section of this paper.

**Figure 2:**
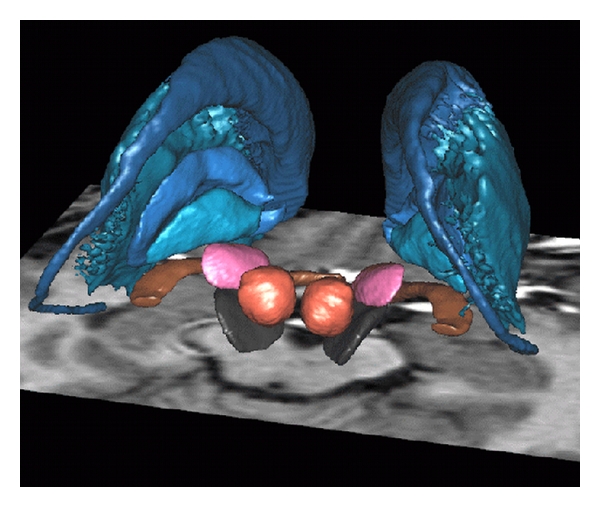
Basal ganglia [13]. In blue, from top to bottom: Caudate nuclei, putamen, external, and internal pallidum. Pink: subthalamic nuclei. Red: red nuclei. Black: substantia nigra. Brown: optic tract.

Subcortical structures might also play a central role in the integration of sensorimotor, limbic, and associative inputs. Yelnik and colleagues [13] have shown that different neural structures constituting the basal ganglia (for instance, the caudate nucleus, the striatum, and of course, the subthalamic nucleus) integrate the sensory-motor, associative, and limbic functional components (Figure 2). This suggests that motor disorders, induced by subcortical dysfunctions at the level of the basal ganglia, could have either a direct or an indirect effect on emotion processes such as the recognition of EFEs through the impairment of motor processing.

## 2. Emotional Impairment Induced by Facial Amimia in Parkinson's Disease

Parkinson's disease (PD) is characterized by the degeneration of dopaminergic neurons in the substantia nigra pars compacta leading to motor as well as associative or addictive disorders [14–16]. The motor disorders characterizing PD are produced by the modulation of neural activity in striatal structures innervated by neurons in substantia nigra pars compacta [17]. The dopaminergic depletion produced by the degeneration of substantia nigra pars compacta induces functional hyperactivity of the subthalamic nuclei and consequently leads to motor disorders that could be modulated by L-Dopa therapy.

Moreover, bilateral deep brain stimulation of the subthalamic nuclei (STN-DBS) is often used as a therapy for these patients. STN-DBS is a well-documented and efficient treatment for severely disabled Parkinson's disease (hereafter, PD) patients with intractable motor complications. Although there is compelling evidence for the clinical efficiency of STN-DBS on motor symptoms [18, 19], there is still an ongoing debate on the effects of STN-DBS on behavioral and emotional manifestations. On one hand, Biseul et al. [20] showed that STN-DBS may produce significant emotional impairment specifically in the recognition of fear. However, on the other hand, Dujardin et al. [21] reported a more extensive impact of STN-DBS for PD patients concerning the recognition of emotional expressions.

These results raise the hypothesis that both L-Dopa and STN-DBS therapies impact on motor as well as emotional behavior. However, in addition to these emotional troubles induced by central disorders, other emotional disorders might indirectly be induced by peripheral dysfunction. Among the different motor disorders of which PD patients suffer, amimia often appears as a consequence of motor symptoms in PD. This motor problem may have consequences for subsequent emotional processes. As proposed elsewhere under the framework of grounded cognition [2, 3, 22, 23], motor disorders could produce emotion recognition impairment. The facial amimia suffered by PD patients induces more severe and long-term deficiencies than temporarily holding a pen in one's mouth during the course of an experiment. We believe that motor disorders may induce a wide range of emotional impairments that could be demonstrated even through emotion recognition tasks.

## 3. Emotional Impairment Induced by Facial TICs in Tourette's Syndrome

As is PD, Tourette's syndrome (TS) is characterized in part by motor disorders. Whereas PD patients often suffer from facial amimia, TS patients often have facial tics. More precisely, TS is a neuropsychiatric syndrome defined by chronic multiple motor and vocal tics sometimes accompanied by anxiety or obsessive-compulsive disorders (OCD). As suggested by Mink [24], basal ganglia and frontocortical dysfunctions probably constitute the root of TS physiopathology (see also [25] for a review). Sprengelmeyer et al. [26] proposed that neurophysiological and neuropsychological data on OCD highlight abnormalities in frontostriatal regions [27] that may potentially mediate correct recognition of emotional expressions.

Another proposal in the pathophysiology of TS is the dopaminergic hypothesis. Albin and Mink [28] suggest a dopaminergic dysregulation that could be at the root of the Tourette's syndrome. This hypothesis is based on biochemical analyses of postmortem striatum from TS patients revealing a significant elevation in the number of dopamine uptake carrier sites [29, 30]. The hypothesis is also derived from animal data that shows that the injection of a dopaminergic agonist increases the production of motor stereotypy very similar to TS in treated animals [31]. Moreover, similar dopaminergic modulations were reported with TS children [32]. Collectively, such dopaminergic sensitivity in TS could be sufficient to induce motor dysfunction (verbal but also motor tics).

Theories of grounded cognition extended to account for emotion processing [3] suggest that motor disorders might produce deficits in the processing of emotional information, as has been previously suggested for Parkinson's disease. That is, as regards Parkinson's disease, dopaminergic dysfunction in the basal ganglia might induce compromised processing of motor functions with consecutive consequences for emotional information processing.

## 4. Theoretical Considerations

Based on the embodiment theory, the motor disorders characterizing Tourette's syndrome (motor hyperactivity and tics) and Parkinson's disease (dyskinesia) should be sufficient to produce emotional disturbance. As suggested by previous findings ([5] see also [3] for a review), motor dysfunction at a peripheral level is sufficient to induce emotional modulation at the central level. Moreover, recent debate shows that such consequences of the embodiment theory on emotional processes could be very fast and beyond the scope of consciousness [33–35]. However, such potential influence of dopaminergic dysfunction on emotional processes through motor disorders could be accompanied by other problems directly affecting the central level.

Concerning Parkinson's disease, it has been shown that dopaminergic depletion has important consequences at the motor level because of the hyperactivity of the subthalamic nuclei [17]. However, as reported by Yelnik et al. [13], the subthalamic nuclei also include the associative and limbic functional components. In other words, dopaminergic depletion could also have a major influence on the limbic and associative functional components indirectly through motor disorders at a peripheral level but also directly at the level central nervous system through subthalamic nuclei hyperactivity. Moreover, dopaminergic depletion may have functional consequences on other neural structures at the level of the basal ganglia, and more specifically on the limbic components of these subcortical structures (as well as related cortical areas). This implies that dopaminergic depletion could have not only a direct influence on emotional processes occurring at the central level but also an indirect influence, based on the embodiment theory, for example, through facial amimia observed at the peripheral level.

Similar conclusions can be drawn for Tourette's syndrome. A dopaminergic hypersensitivity as proposed by [31] or [29] might have important consequences on emotional processes occurring at the level of the basal ganglia. The pallidum, a subcortical neural structure potentially involved in TS [28], could have direct implications on emotional processes [13] in a manner similar to subthalamic nuclei in PD. As for PD patients, dopaminergic modulation probably has a direct influence on basal ganglia (and, therefore, subcortical limbic processes) combined with an indirect influence through the well-established motor disorders observed in TS (still under the theoretical framework of the embodiment theory). However, despite these strong theoretical assumptions of emotional disorders in PD and TS, literature has brought into light controversial evidence concerning emotional impairments that recommends to carefully take into account methodological considerations.

## 5. Methodological Considerations

An examination of the literature on emotional processing in PD yields a set of controversial findings. For instance, Sprengelmeyer et al. [36] reported that recent untreated PD patients presented significant EFE impairments (more specifically on disgust) as compared to healthy controls but this difference was not observed on more severe but treated PD patients. These findings were corroborated by the study of Suzuki et al. [37]. Using an innovative technique allowing to take into account the difficulty of recognizing one or another EFE, the authors reported significant impairments on disgust EFE. However, using an experimental procedure for recording accuracy in categorizing EFE together with a rating scale of the intensity of each EFE, Dujardin et al. [21] reported more widespread emotional impairments compared to previous studies. They found that PD patients were less accurate than healthy controls in decoding angry, sad, and disgusted EFEs. Moreover, PD patients rated EFEs other than anger, sadness, and disgust as less intense than healthy controls. More surprisingly, PD patients rated the surprise EFE more intensely than the healthy control group. Generally, these last findings suggest that Parkinson's disease patients may have more widespread emotional impairments than previously reported in literature, but the experimental tasks habitually undertaken were not sensitive enough to reveal these impairments. In other words, PD patients might use perceptual strategies to overcome emotional impairments. Consistent with this interpretation, Adolphs et al. [38] reported interesting data showing that a patient with severe amygdala lesion inducing a strong deficit in the *feeling* of fear was nonetheless able to recognize in a reliable manner fearful expressions by looking at specific perceptual details in the face such as the opening of the eyes. This experiment illustrates the fact that participants may use *perceptual* details, even without a clear emotional feeling of the different EFEs, to recognize EFEs. In order to decrease the influence of perceptual strategies on the performance of EFE tasks, we advocate the use of rapid presentations of emotional stimuli. Rapid presentation of critical stimuli, such as EFEs, reduces the use of perceptual strategies that can mask the existence of specific emotional deficits such that they go undetected in studies with longer presentation durations [21, 36, 37]. 

In their examination of Tourette's syndrome, Sprengelmeyer et al. [26] found that TS patients with OCD have a specific impairment in recognizing disgusted faces. This impairment was not observed in TS patients without OCD. Similar results were obtained by means of fMRI experiments by Shapira et al. [39]. They showed different distributions of brain activation after disgust-inducing visual stimulation for OCD patients compared to control participants whereas activation after threat-inducing stimulation in OCD participants induced a similar pattern to that observed in control participants. However, Parker et al. [40] failed to replicate the finding of Sprengelmeyer et al. [26] and found only a disgust impairment for one TS patient with severe OCD. However, as for PD patients, the sensitivity of the task may not be sufficient to show clear evidence of emotional impairment. In both Sprengelmeyer et al. [26] and Parker et al. [40], TS participants were exposed to morphed stimuli (from one EFE to another one) across the emotion hexagon of the 6 basic EFE produced by Ekman and Friesen's [41]. The advantage is to repeatedly expose participants to continuous variations of the same EFE. However, a potential disadvantage is that such repeated exposure to the morphed stimuli across the hexagon significantly improves the perceptual participant's ability to categorize the EFEs, and this might be sufficient to induce a ceiling effect. More importantly, long and repeated exposure durations (1000 to 5000 ms stimulus duration) could enable the participants to use cognitive strategies based on specific perceptual features to perform the task. As for PD experiments, these studies illustrate the fact that participants may use perceptual details, even without a clear emotional feeling of the different EFEs, to perform the task.

## 6. Conclusion

This theoretical paper highlights different consequences of grounded cognition literature. Based on this literature, we can assume that the motor disorders of which PD and TS patients suffer probably imply important consequences at the emotional level, through the disruption of the embodied processing of emotional events. So far, based on the experimental designs used in previous simulations, we can assume that the absence of reliable results showing such deficits could probably be due to perceptual strategies potentially compensating for the emotional troubles of the patients. Therefore, further research will have to (i) show such emotional deficits based on more implicit measures of emotional feelings and (ii) differentiate between deficits induced by dopaminergic dysregulation occurring at the central level and embodied processing disorders occurring at the peripheral level.

## References

[1] Bower GH (1981). Mood and memory. *American Psychologist*.

[2] Barsalou LW (1999). Perceptual symbol systems. *Behavioral and Brain Sciences*.

[3] Niedenthal PM (2007). Embodying emotion. *Science*.

[4] Niedenthal PM, Mermillod M, Maringer M, Hess U (2010). The future of SIMS: who embodies which smile and when?. *Behavioral and Brain Sciences*.

[5] Strack F, Martin LL, Stepper S (1988). Inhibiting and facilitating conditions of the human smile: a nonobtrusive test of the facial feedback hypothesis. *Journal of Personality and Social Psychology*.

[6] Gallese V, Fadiga L, Fogassi L, Rizzolatti G (1996). Action recognition in the premotor cortex. *Brain*.

[7] Rizzolatti G, Fadiga L, Gallese V, Fogassi L (1996). Premotor cortex and the recognition of motor actions. *Cognitive Brain Research*.

[8] Decety J, Grèzes J (1999). Neural mechanisms subserving the perception of human actions. *Trends in Cognitive Sciences*.

[9] Wicker B, Keysers C, Plailly J, Royet JP, Gallese V, Rizzolatti G (2003). Both of us disgusted in my insula: the common neural basis of seeing and feeling disgust. *Neuron*.

[10] Adolphs R, Damasio H, Tranel D, Damasio AR (1996). Cortical systems for the recognition of emotion in facial expressions. *Journal of Neuroscience*.

[11] Adolphs R, Damasio H, Tranel D, Cooper G, Damasio AR (2000). A role for somatosensory cortices in the visual recognition of emotion as revealed by three-dimensional lesion mapping. *Journal of Neuroscience*.

[12] Adolphs R (2002). Recognizing emotion from facial expressions: psychological and neurological mechanisms. *Behavioral and Cognitive Neuroscience Reviews*.

[13] Yelnik J, Bardinet E, Dormont D (2007). A three-dimensional, histological and deformable atlas of the human basal ganglia. I. Atlas construction based on immunohistochemical and MRI data. *NeuroImage*.

[14] Doshi PK, Chhaya N, Bhatt MH (2002). Depression leading to attempted suicide after bilateral subthalamic nucleus stimulation for Parkinson’s disease. *Movement Disorders*.

[15] Houeto JL, Mesnage V, Mallet L (2002). Behavioral disorders, Parkinson’s disease and subthalamic nucleus. *Journal of Neurology, Neurosurgery and Psychiatry*.

[16] Ulla M, Thobois S, Lemaire JJ (2006). Manic behaviour induced by deep-brain stimulation in Parkinson’s disease: evidence of substantia nigra implication?. *Journal of Neurology, Neurosurgery and Psychiatry*.

[17] Parent A (1990). Extrinsic connections of the basal ganglia. *Trends in Neurosciences*.

[18] Derost P, Ouchchane L, Morand D (2007). Is DBS-STN appropriate to manage severe Parkinson’s disease in an elderly population?. *Neurology*.

[19] Limousin P, Krack P, Pollak P (1998). Electrical Stimulation of the Subthalamic Nucleus in Advanced Parkinson’s Disease. *The New England Journal of Medicine*.

[20] Biseul I, Sauleau P, Haegelen C (2005). Fear recognition is impaired by subthalamic nucleus stimulation in Parkinson’s disease. *Neuropsychologia*.

[21] Dujardin K, Blairy S, Defebvre L (2004). Deficits in decoding emotional facial expressions in Parkinson’s disease. *Neuropsychologia*.

[22] Dimberg U (1990). Facial electromyography and emotional reactions. *Psychophysiology*.

[23] Hess U, Blairy S (2001). Facial mimicry and emotional contagion to dynamic emotional facial expressions and their influence on decoding accuracy. *International Journal of Psychophysiology*.

[24] Mink JW (2001). Basal ganglia dysfunction in Tourette’s syndrome: a new hypothesis. *Pediatric Neurology*.

[25] Osmon DC, Smerz JM (2005). Neuropsychological evaluation in the diagnosis and treatment of Tourette’s syndrome. *Behavior Modification*.

[26] Sprengelmeyer R, Young AW, Pundt I (1997). Disgust implicated in obsessive-compulsive disorder. *Proceedings of the Royal Society B*.

[27] Abbruzzese M, Ferri S, Scarone S (1997). The selective breakdown of frontal functions in patients with obsessive-compulsive disorder and in patients with schizophrenia: a double dissociation experimental finding. *Neuropsychologia*.

[28] Albin RL, Mink JW (2006). Recent advances in Tourette syndrome research. *Trends in Neurosciences*.

[29] Singer HS, Walkup JT (1991). Tourette syndrome and other tic disorders. Diagnosis, pathophysiology, and treatment. *Medicine*.

[30] Singer HS, Hahn IH, Moran TH (1991). Abnormal dopamine uptake sites in postmortem striatum from patients with Tourette’s syndrome. *Annals of Neurology*.

[31] Delfs JM, Kelley AE (1990). The role of D1 and D2 dopamine receptors in oral stereotypy induced by dopaminergic stimulation of the ventrolateral striatum. *Neuroscience*.

[32] Ernst M, Zametkin AJ, Jons PH, Matochik JA, Pascualvaca D, Cohen RM (1999). High presynaptic dopaminergic activity in children with Tourette’s disorder. *Journal of the American Academy of Child and Adolescent Psychiatry*.

[33] Vermeulen N, Godefroid J, Mermillod M (2009). Emotional modulation of attention: fear increases but disgust reduces the attentional blink. *PLoS ONE*.

[34] Vermeulen N, Mermillod M (2010). Fast emotional embodiment can modulate sensory exposure in perceivers. *Communicative and Integrative Biology*.

[35] Vermeulen N, Mermillod M, Godefroid J, Corneille O (2009). Unintended embodiment of concepts into percepts: sensory activation boosts attention for same-modality concepts in the attentional blink paradigm. *Cognition*.

[36] Sprengelmeyer R, Young AW, Mahn K (2003). Facial expression recognition in people with medicated and unmedicated Parkinson’s disease. *Neuropsychologia*.

[37] Suzuki A, Hoshino T, Shigemasu K, Kawamura M (2006). Disgust-specific impairment of facial expression recognition in Parkinson’s disease. *Brain*.

[38] Adolphs R, Gosselin F, Buchanan TW, Tranel D, Schyns P, Damasio AR (2005). A mechanism for impaired fear recognition after amygdala damage. *Nature*.

[39] Shapira NA, Liu Y, He AG (2003). Brain activation by disgust-inducing pictures in obsessive-compulsive disorder. *Biological Psychiatry*.

[40] Parker HA, McNally RJ, Nakayama K, Wilhelm S (2004). No disgust recognition deficit in obsessive-compulsive disorder. *Journal of Behavior Therapy and Experimental Psychiatry*.

[41] Ekman P, Friesen WV (1976). *Pictures of Facial Affect*.

